# The Adaptive Immune Landscape of the Colorectal Adenoma–Carcinoma Sequence

**DOI:** 10.3390/ijms22189791

**Published:** 2021-09-10

**Authors:** João Augusto Freitas, Irene Gullo, Diogo Garcia, Sara Miranda, Louisa Spaans, Lídia Pinho, Joana Reis, Fabiana Sousa, Manuela Baptista, Carlos Resende, Dina Leitão, Cecília Durães, José Luis Costa, Fátima Carneiro, José Carlos Machado

**Affiliations:** 1 Instituto de Investigação e Inovação em Saúde (i3S), University of Porto, 4200-135 Porto, Portugal; joaoaugusto2freitas@gmail.com (J.A.F.); irene.gullo12@gmail.com (I.G.); sara.csm_94@hotmail.com (S.M.); lpinho@i3s.up.pt (L.P.); joana.reis@ibmc.up.pt (J.R.); cresende@ipatimup.pt (C.R.); cduraes@ipatimup.pt (C.D.); jcosta@ipatimup.pt (J.L.C.); fcarneiro@ipatimup.pt (F.C.); 2 Institute of Molecular Pathology and Immunology, University of Porto (Ipatimup), 4200-135 Porto, Portugal; 3 Department of Pathology, Centro Hospitalar Universitário de São João (CHUSJ), 4200-319 Porto, Portugal; 4 Department of Pathology, Faculty of Medicine of the University of Porto (FMUP), 4200-319 Porto, Portugal; diogo.monizgarcia@gmail.com (D.G.); dinaraquel@med.up.pt (D.L.); 5 Faculty of Health, Medicine and Life Sciences-Maastricht University, 6229 ER Maastricht, The Netherlands; l.spaans@student.maastrichtuniversity.nl; 6 High Risk Consultation of Digestive Tumors, Department of General Surgery, Centro Hospitalar Universitário de São João (CHUSJ), 4200-319 Porto, Portugal; fabiana.s.sousa52@gmail.com (F.S.); manuela.balsinha@sapo.pt (M.B.)

**Keywords:** colorectal adenoma, colorectal adenocarcinoma, familial adenomatous polyposis, APC germline alterations, tumor immune microenvironment, immunogenicity, PD-L1 expression

## Abstract

Background. The tumor immune microenvironment exerts a pivotal influence in tumor initiation and progression. The aim of this study was to analyze the immune context of sporadic and familial adenomatous polyposis (FAP) lesions along the colorectal adenoma–carcinoma sequence (ACS). Methods. We analyzed immune cell counts (CD3+, CD4+, CD8+, Foxp3+, and CD57+), tumor mutation burden (TMB), MHC-I expression and PD-L1 expression of 59 FAP and 74 sporadic colorectal lesions, encompassing adenomas with low-grade dysplasia (LGD) (30 FAP; 30 sporadic), adenomas with high-grade dysplasia (22 FAP; 30 sporadic), and invasive adenocarcinomas (7 FAP; 14 sporadic). Results. The sporadic colorectal ACS was characterized by (1) a stepwise decrease in immune cell counts, (2) an increase in TMB and MHC-I expression, and (3) a lower PD-L1 expression. In FAP lesions, we observed the same patterns, except for an increase in TMB along the ACS. FAP LGD lesions harbored lower Foxp3+ T cell counts than sporadic LGD lesions. A decrease in PD-L1 expression occurred earlier in FAP lesions compared to sporadic ones. Conclusions. The colorectal ACS is characterized by a progressive loss of adaptive immune infiltrate and by the establishment of a progressively immune cold microenvironment. These changes do not appear to be related with the loss of immunogenicity of tumor cells, or to the onset of an immunosuppressive tumor microenvironment.

## 1. Introduction

The etiopathogenesis of most colorectal carcinomas (CRC) follows the conventional adenoma–carcinoma sequence (ACS), in which colorectal adenomas with low-grade dysplasia (LGD) and/or high-grade dysplasia (HGD) evolve to invasive adenocarcinoma (ADC), through a stepwise accumulation of genetic and epigenetic alterations. The earliest events in colorectal tumorigenesis involve alterations of the WNT signaling pathway, most frequently through somatic mutation of the *adenomatous polyposis coli* (*APC*) gene [1,2,3]. Germline mutations in the *APC* gene are the genetic cause of familial adenomatous polyposis (FAP), an autosomal dominant syndrome characterized by numerous adenomatous lesions in the colorectum, and early onset CRC.

The tumor immune microenvironment has a crucial role in the development and progression of colorectal precursor and invasive lesions [4]. Moreover, the amount, type, and location of tumor-infiltrating lymphocytes have been shown to have prognostic value in CRC patients [5,6,7]. With the widespread use of targeted immunotherapy in solid tumors, including CRC [8], there is a growing research interest in exploring the role of the tumor immune microenvironment also as a predictive biomarker. Currently, monoclonal antibodies against immune checkpoints, e.g., CTLA4, PD-1, and PD-L1, have been approved in microsatellite instability-high (MSI-H) CRCs and microsatellite-stable (MSS) tumors with high tumor mutation burden (TMB) [9,10], reflecting the immunogenicity of these tumor subtypes [11].

In the current body of literature, great attention has been given to the immune landscape of invasive ADC, whereas few studies have compared the tumor immune microenvironment of pre-invasive and malignant lesions, with contradictory findings [4,12,13,14,15,16]. Some studies have demonstrated a lower expression of Th1 cytokines, a shift to a Th2 cytokine profile [4,15], and a decrease in cytotoxic CD8+ T cell counts along ACS [17,18], whereas others have suggested an increase in the density of T lymphocytes, regulatory T cells, and macrophages during tumor progression [4,14,16]. Likewise, little information is available regarding the comparison of human colorectal neoplastic lesions arising from a sporadic or hereditary background, since most studies on hereditary cancer have used mouse models, especially *APCmin* mice [19]. Furthermore, most of the studies in human colorectal cancer focused on MSI tumors. A study by Liu, G. et al. showed that the number of CD3+, CD8+, CD4+ and FoxP3+ cells in the invasive margin or tumor stroma is higher in Lynch syndrome cases than in sporadic cases [20]. Therefore, an in-depth characterization of the human tumor immune landscape and its evolution along the colorectal ACS is needed, especially for MSS colorectal cancer.

The present study represents the first attempt to analyze the immune context of sporadic and hereditary FAP-related lesions along the colorectal ACS. We analyzed the density, quality, and temporal distribution of tumor-infiltrating immune cells (CD3+, CD4+, CD8+, Foxp3+, and CD57+), as well as molecular and immunophenotypic biomarkers of tumor immunogenicity, along the development of colorectal precursor and invasive lesions.

## 2. Results

**Figure 1:**
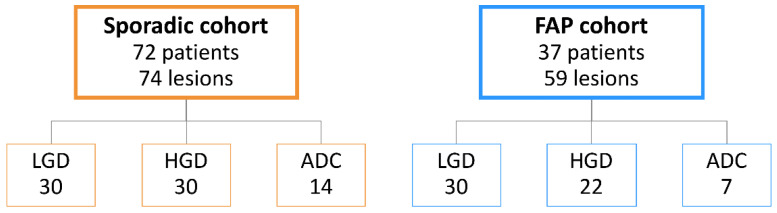
Cohort characterization, encompassing sporadic and Familial Adenomatous Polyposis (FAP) lesions. LGD, low-grade dysplasia; HGD, high-grade dysplasia; ADC, invasive adenocarcinoma.

The cohort analyzed in this study encompasses 37 patients with FAP (21 males and 16 females) and 72 patients harboring sporadic lesions (43 males and 29 females). FAP lesions were diagnosed at the median age of 44 years, as compared to 63 years of age of patients with sporadic lesions. For each group of patients, we analyzed colorectal adenomas with LGD (*n* = 30 FAP and *n* = 30 sporadic), with HGD (*n* = 22 FAP and *n* = 30 sporadic) and colorectal invasive ADC (*n* = 7 FAP and *n* = 14 sporadic) (Figure 1). The presence of mismatch repair protein deficiency (MMRP-D) and high-status MSI were excluded in invasive ADC from the sporadic cohort.

The clinicopathological characteristics of colorectal ADC cases included in this study are described in Table 1.

### 2.1. Tumor Infiltration with Immune Cells Decreases throughout the Colorectal ACS

To investigate the evolution of the tumor immune microenvironment along the colorectal ACS, we analyzed the density and quality of the immune infiltration in adenomatous lesions with LGD, with HGD, and in invasive ADCs. By immunohistochemistry and counting representative areas, we analyzed and normalized the density of the overall population of CD3+ T lymphocytes per mm^2^, as well as the specific populations of CD4+ helper T cells, cytotoxic CD8+ T lymphocytes, Foxp3+ regulatory T cells, and CD57+ T lymphocytes/natural killer (NK) cells.

**Figure 2:**
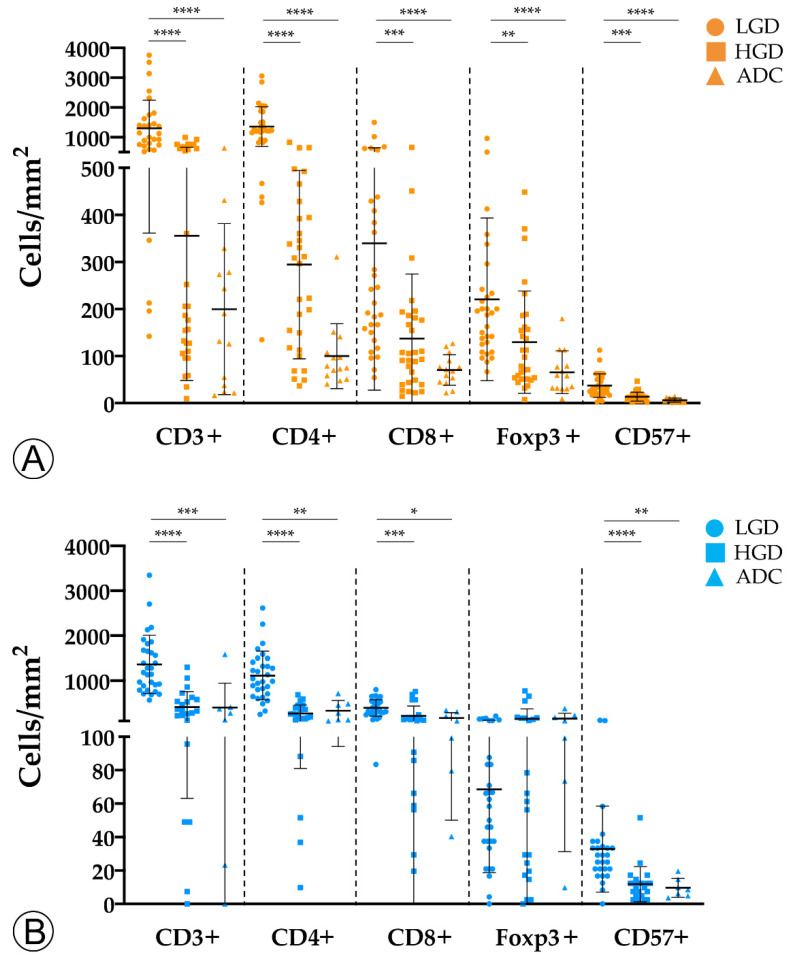
Counts of immune cells along the colorectal adenoma-carcinoma sequence in sporadic (**A**) and hereditary Familial Adenomatous Polyposis (FAP)-related (**B**) lesions. LGD, low-grade dysplasia; HGD, high-grade dysplasia; ADC, invasive adenocarcinoma; * = *p* < 0.05; ** = *p* < 0.01; *** = *p* < 0.001; **** = *p* < 0.0001.

We observed an overall decrease in tumor-infiltrating immune cells along the colorectal ACS in both sporadic and hereditary FAP-related cohorts (Figure 2). Adenomatous lesions with LGD harbored higher counts of CD3+, CD4+, CD8+, and CD57+ immune cells when compared with adenomatous lesions with HGD and invasive ADCs, in both sporadic and hereditary FAP-related lesions. These findings were more pronounced in sporadic lesions, with *p*-values < 0.001 when adenomatous lesions with LGD were compared with ADCs. Although the density of Foxp3+ regulatory T cells followed the same decrease pattern in the sporadic context, this was not observed in lesions from FAP patients. As the spatial distribution of immune cell types within the tumors could be relevant in the interpretation of our results, we studied the immune cell density separately at the center and at the front of invasive ADCs. No differences were found between the two locations (data not shown) and the results on immune cell counts were considered as a whole.

**Figure 3:**
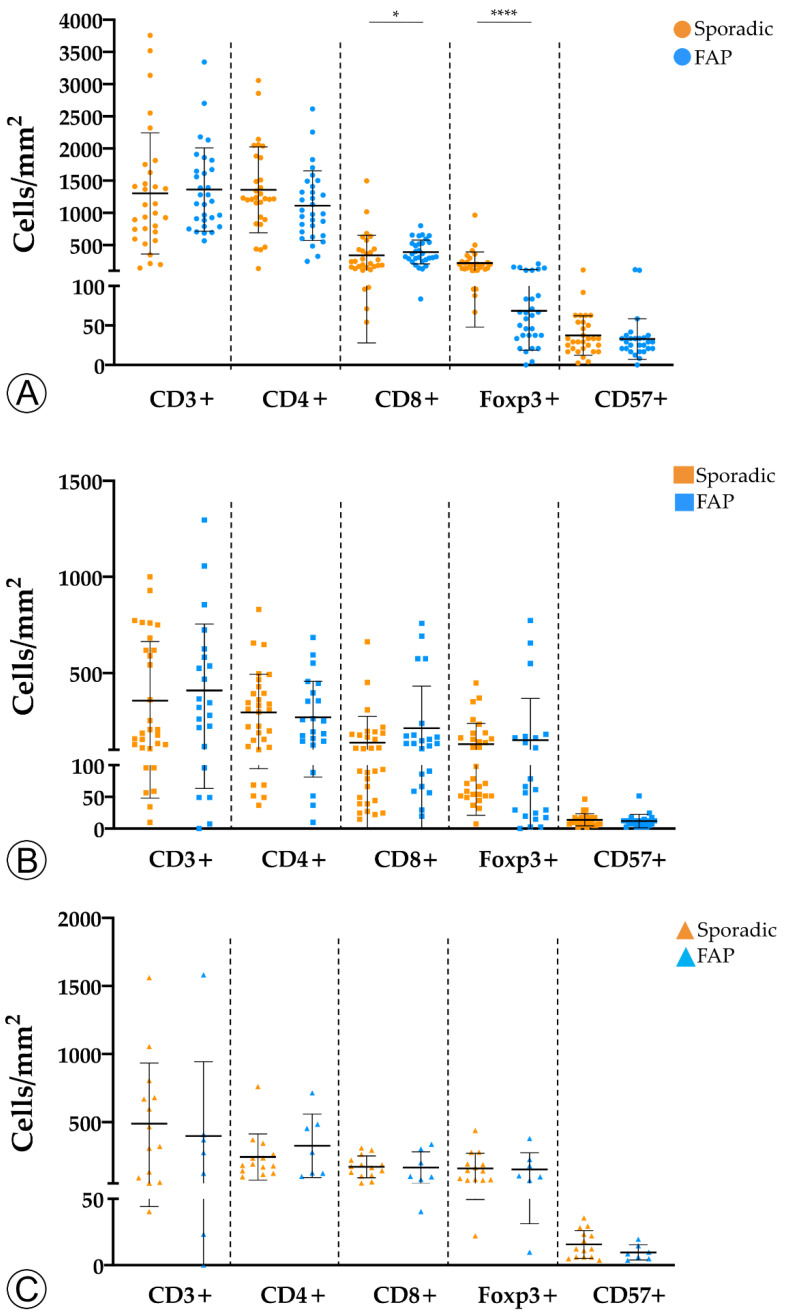
Comparison of immune cell counts between sporadic and hereditary Familial Adenomatous Polyposis (FAP)-related lesions in adenomatous lesions with low-grade dysplasia (**A**), adenomatous lesions with high-grade dysplasia (**B**), and invasive adenocarcinoma (**C**). * = *p* < 0.05; **** = *p* < 0.0001.

To assess whether the tumor immune microenvironment could vary depending on the hereditary or sporadic background of the neoplastic lesions, we investigated the immune landscape between the two cohorts for LGD, HGD, and invasive ADC lesions (Figure 3).

We found that adenomas with LGD from FAP patients displayed a marginal increase in CD8+ T cells (*p* < 0.05) and a significantly lower density of Foxp3+ (*p* < 0.0001) T cells when compared with sporadic adenomas with LGD. No differences were observed for the remaining adaptive immune cell populations, or subgroups of sporadic and hereditary lesions.

In conclusion, our findings suggest that colorectal neoplastic lesions trigger a host immune response, but become immunologically colder throughout the colorectal ACS. This was observed for both sporadic and hereditary lesions, although it was more pronounced in the former. In that regard, we would like to stress the higher density of Foxp3+ regulatory T cells in sporadic versus hereditary LGD lesions, suggesting that the host has a higher baseline immunological tolerance towards hereditary lesions.

### 2.2. TMB and MHC-I Expression Increase along the Colorectal ACS

We hypothesized that the decrease in immune cells counts along the ACS could be partially explained by the progressive loss of immunogenicity [23,24]. To explore this hypothesis, we evaluated TMB and the expression of the major histocompatibility complex class I (MHC-I) protein in tumor cells as surrogates to tumor antigenicity, and to the ability of tumor cells to present neoantigens to the immune system, respectively.

**Figure 4:**
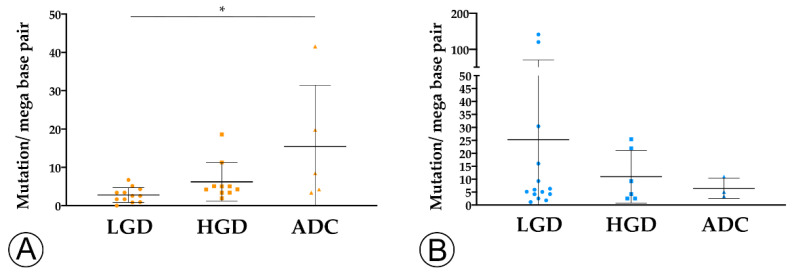
TMB analysis along the colorectal adenoma-carcinoma in sporadic (**A**) and hereditary Familial Adenomatous Polyposis (FAP)-related (**B**) lesions. * = *p* < 0.05. LGD, low-grade dysplasia (circles); HGD, high-grade dysplasia (squares); ADC, invasive adenocarcinoma (triangles); * = *p* < 0.05.

**Figure 5:**
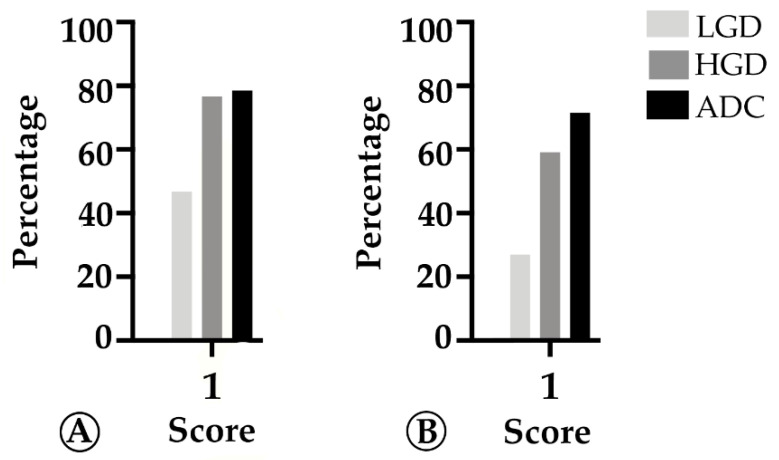
Percentage of cases in which MHC-I in tumor cells is >25% (score 1) along the colorectal adenoma-carcinoma sequence, in sporadic (**A**) and hereditary Familial Adenomatous Polyposis (FAP)-related (**B**) lesions. LGD, low-grade dysplasia; HGD, high-grade dysplasia; ADC, invasive adenocarcinoma.

**Figure 6:**
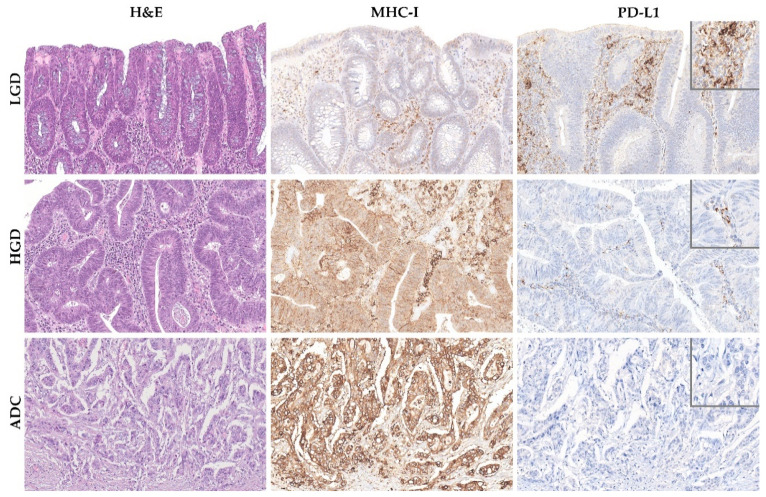
MHC-I and PD-L1 expression along the colorectal adenoma-carcinoma squence in representative examples of sporadic adenomatous lesion with low-grade dysplasia (LGD) (**upper** panel), sporadic adenomatous lesion with high-grade dysplasia (HGD) (**middle** panel), and sporadic invasive adenocarcinoma (ADC) (**lower** panel). Note the membranous and cytoplasmic immunoreactivity of MHC-I in neoplastic cells of HGD and invasive ADC lesions. Hematoxylin and eosin (**left** panel) and immunohistochemistry for MHC-I (**middle** panel) and PD-L1 (**right** panel), 40× amplification. The insets in the right panel represent higher magnification of PD-L1 immunohistochemistry, 400× amplification.

We found that TMB increased throughout the ACS in sporadic cases (Figure 4A), from the median value of 2.76 mutation/mega base pair (mut/Mbp) in LGD lesions to 15.76 mut/Mbp in ADC cases (*p* < 0.05). In contrast, this trend was not observed in hereditary FAP-related lesions (Figure 4B), where the average number of mut/Mbp remained constant along the ACS, despite the presence of some outlier samples. Likewise, we found that MHC-I expression in neoplastic cells progressively increased throughout the ACS in both FAP-related (*p* = 0.033) and sporadic (*p* = 0.025) lesions (Figure 5). MHC-I was expressed at the cell membrane, as well as in the cytoplasm of neoplastic cells (Figure 6).

Overall, the increase in TMB in sporadic lesions, and the increase in MHC-I expression in both sporadic and FAP lesions along the ACS suggest that the observed decrease in immune cell counts cannot be explained by a loss of immunogenicity.

### 2.3. PD-L1 Expression Decreases throughout the Colorectal Adenoma Carcinoma Sequence

To verify whether the decrease in adaptive immune cell counts could be explained by the establishment of an immunosuppressive microenvironment, we analyzed the expression of the PD-L1 immune checkpoint protein in tumor stroma cells [25]. Among the different immune checkpoint proteins available, PD-L1 was chosen because it is the most widespread used predictive biomarker of response to immunotherapy.

**Figure 7:**
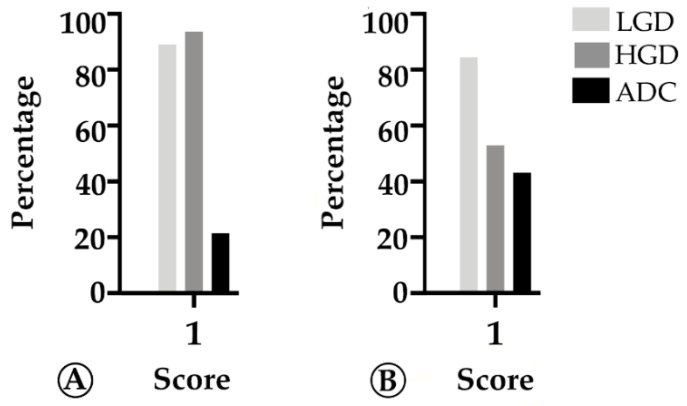
Percentage of cases in which PD-L1 expression in tumor stroma cells is >1% (score 1) along the colorectal adenoma-carcinoma sequence, in sporadic (**A**) and hereditary Familial Adenomatous Polyposis (FAP)-related (**B**) lesions. LGD, low-grade dysplasia; HGD, high-grade dysplasia; ADC, invasive adenocarcinoma.

The pattern of expression was mostly membranous in stroma immune cells (Figure 6), and focally in tumor cells in rare cases. Our findings revealed that PD-L1 expression progressively decreased along the ACS in both FAP-related (*p* = 0.028) and sporadic (*p* < 0.001) lesions (Figure 7). These findings, coupled with the progressive decrease in Foxp3+ immune cell counts along the colorectal ACS, indicate that there is no establishment of an immunosuppressive microenvironment during colorectal tumor progression.

## 3. Discussion

The ability of precancerous lesions to progress to cancer is related not only to the intrinsic phenotypic or molecular traits of neoplastic cells, but also to the contributions of mesenchymal and immune cells constituting the so-called tumor microenvironment [26]. A few studies have explored the immune microenvironment of colorectal lesions by comparing adenomatous polyps with invasive ADCs, and the findings of these studies are controversial. In some studies, an increase in the density of T lymphocytes, regulatory T cells, and macrophages during tumor progression was observed [4,14,16], while in others, a decrease in cytotoxic CD8+ T cell counts from adenomatous lesions to ADCs [17,18], as well as a lower expression of Th1 cytokines and a shift to a Th2 cytokine profile, have been reported [4,15]. Despite being poorly explored, the characterization of the tumor microenvironment along the ACS is crucial to understanding colorectal cancer initiation and progression. In the present study, we aimed to characterize the population of tumor-infiltrating immune cells (CD3+, CD4+, CD8+, Foxp3+, and CD57+) along the ACS, and to uncover potential differences between adenomatous and invasive lesions in the sporadic setting versus the hereditary (FAP-related) context.

Supporting the most recent findings in the literature [17], our findings suggest that colorectal carcinogenesis is characterized by the gradual loss of adaptive immune cell counts along the colorectal ACS, in both sporadic and hereditary lesions. Many factors may contribute to our findings [27,28,29,30,31]. Among them, one of the most well-recognized mechanisms is the reduction in neoantigen presentation by tumor cells, leading to an impairment of the adaptive immune response [27,32]. To address this, we investigated TMB, as well as the expression of MHC-I on the surface of tumor cells. Despite a decrease in immune cell counts along the colorectal ACS, we observed higher TMB and MHC-I expression along colorectal tumor progression, which is a finding that does not support the loss of immunogenicity contention.

To evaluate whether an immune-suppressive tumor microenvironment may represent an alternative mechanism to explain the lower density of adaptive immune cells along the ACS, we analyzed PD-L1 expression in tumor stroma cells. Our results did not support the establishment of an immunosuppressive microenvironment, as PD-L1 expression decreased along the colorectal ACS. These findings are also corroborated by the progressive decrease in Foxp3+ regulatory T cell counting along the colorectal ACS. Although our findings did not demonstrate an increase in immune-suppressive biomarkers along the ACS, we may not exclude the influence of other types of immune cells, which have not been analyzed in this study, e.g., innate immune cells or B cells. Indeed, some studies support the importance of other immune cell types for tumor development [33,34,35].

Regarding the comparison of the immune microenvironment between sporadic and hereditary FAP-related lesions, we observed a significantly lower density of Foxp3+ regulatory T cells in FAP adenomas with LGD, as compared with sporadic adenomas with LGD. These results lead us to speculate that neoplastic cells arising in a hereditary background may be less prone to trigger an immune response, and are in line with those reported by Majumder S. et al., who showed that CD8+ T cells are not activated by tumor cells in an FAP background [36].

A limitation of the current study is its “static” nature, which reflects the quantity and quality of the immune cells in the lesions, but not their activation status. Nevertheless, our study sheds light on the interplay between the immune system and the ACS, which assumes particular relevance, considering the progressive increase in immunotherapy strategies for colorectal cancer patients. Similar analysis in other cohorts of patients, as well as analysis of other immune cell types (B-cells, macrophages, etc.), cytokine and chemokine immune profiling, and mRNA sequencing of immune-related genes should be pursued to expand our understanding of the immune landscape along the ACS.

Overall, our findings suggest that the colorectal ACS is characterized by a progressive loss of adaptive immune infiltrate and by the establishment of a progressively immune cold microenvironment. These changes do not appear to be related with the loss of immunogenicity of tumor cells, or with the onset of an immunosuppressive tumor microenvironment. The progressive loss of all types of adaptive immune cells rather suggests a dysfunctional state of immune cell exhaustion that likely results from persistent antigen stimulation and chronic inflammation.

## 4. Materials and Methods

### 4.1. Patient Series

Formalin fixed paraffin-embedded (FFPE) samples were collected retrospectively from the Department of Pathology of Centro Hospitalar Universitário de São João (CHUSJ, Porto, Portugal). The cohort of patients included 37 FAP patients, diagnosed at our center on the basis of family/personal history and genetic analysis, and 72 patients harboring sporadic colorectal lesions. The FAP cohort included 30 adenomas with LGD, 22 adenomas with HGD and 7 invasive ADCs. The sporadic cohort included 30 adenomas with LGD, 30 adenomas with HGD and 14 invasive ADCs. Histopathological analysis was reviewed on hematoxylin and eosin (H&E)-stained slides by two gastrointestinal pathologists (IG, FC). None of the cases included in this study had been previously treated with chemotherapy or radiation therapy.

### 4.2. Immunohistochemistry and Scoring

Serial 3-μm sections were prepared from one representative FFPE block. IHC staining was performed with antibodies against CD3 (clone 2GV6, 1:500) (Ventana Medical Systems, Oro Valley, AZ, USA), CD8 (clone SP57, prediluted) (Ventana Medical Systems, Oro Valley, AZ, USA), CD4 (clone SP35, prediluted) (Ventana Medical Systems, Oro Valley, AZ, USA), CD57 (clone ab187274, 1:300) (Abcam, Cambridge, UK); Foxp3 (clone ab200034, 1:100) (Abcam, Cambridge, UK), PD-L1 (clone 22C3, 1:100) (DAKO, Santa Clara, CA, USA) and MHC-I (clone sc-55582, 1:100) (Santa Cruz Biotechnology, Dallas, TX, USA). Samples were processed in the automatic Ventana Benchmark Ultra platform (Ventana Medical Systems, Oro Valley, AZ, USA) using an Optiview Universal DAB detection kit (Ventana Medical Systems, Oro Valley, AZ, USA) and an Optiview amplification kit (Ventana Medical Systems, Oro Valley, AZ, USA) for PD-L1 staining. Immunohistochemistry for MMR proteins was performed in ADC samples from the sporadic cohort (*n* = 14) with antibodies against MLH1 (clone M1, prediluted) (Ventana Medical Systems, Oro Valley, AZ, USA), MSH2 (clone G219-1129, prediluted) (Ventana Medical Systems, Oro Valley, AZ, USA), MSH6 (clone SP93, prediluted) (Ventana Medical Systems, Oro Valley, AZ, USA)) and PMS2 (clone A16-4, prediluted) (Ventana Medical Systems, Oro Valley, AZ, USA), using the same equipment.

Full-thickness consecutive sections, stained for H&E, and CD3, CD8, CD4, CD57 and Foxp3 proteins were scanned with a 40× objective, using a Nanozoomer S60 slide scanner (Hamamatsu, Hamamatsu city, Japan). The scanned slides were observed using NDP.view2 viewing software (Hamamatsu, Hamamatsu city, Japan) and, for each lesion, two representative fields at 400× magnification were selected by a pathologist (IG) using the snapshot function. Individual cells were manually counted by two observers in a double-blind setting using ImageJ software (National institutes of Health, Bethesa, MD, USA) with the aid of the Cell Counter plugin. The entire images were used for counting and the total area analyzed was normalized to 1 mm^2^.

PD-L1 and MHC-I expression were assessed by a pathologist (IG). PD-L1 was assessed only in immune cells and was considered positive (score 1) when ≥1% immune cells showed immunoreactivity. MHC-I was assessed in neoplastic cells and was considered positive (score 1) when at least 25% of neoplastic cells showed membranous and cytoplasmic immunoreactivity.

MMRP immunoreactivity was classified as (1) intact, when ≥10% of the tumor cells showed preserved nuclear expression, or (2) abnormal expression, when the tumor showed complete loss of expression, expression in <10% of tumor cells [37], weaker staining compared with the internal control, or abnormal staining in the nucleoli or nuclear membrane. All ADCs belonging to the sporadic cohort showed intact expression of MMRPs and were classified as MMRP-proficient.

### 4.3. MSI Testing

MSI phenotype was determined using the Idylla™ MSI test (Biocartis NV, Mechelen, Belgium), as previously described [38], in the ADC samples from the sporadic cohort (*n* = 14). Only MSS cases were included in the study.

### 4.4. Tumor Mutation Burden

DNA was extracted from FFPE samples using the MagMAX™ FFPE DNA/RNA Ultra kit (Thermo Fisher Scientific, Waltham, MA, USA). Manufacturer protocol was followed except for the magnetic bead drying step that was performed at RT for 4–6 min, instead of shaking for 2 min.

The Oncomine™ tumor mutation load assay (Thermo Fisher Scientific, Waltham, MA, USA) was used to determine tumor mutation burden, as instructed by the manufacturer. Library quantification was performed by quantitative PCR using the Ion Library TaqMan quantitation kit (Thermo Fisher Scientific, Waltham, MA, USA).). The Ion Chef system was used for template preparation and chip loading before sequencing using the Ion S5™ XL sequencer (Thermo Fisher Scientific, Waltham, MA, USA). The data were analyzed with Ion Reporter™ software version 5.18.0.1 (Thermo Fisher Scientific, Waltham, MA, USA), and tumor mutation burden was determined as number of mut/Mbp using variants with a minimum of 10% allelic frequency.

### 4.5. Statistical Analysis

Statistical analysis was performed using GraphPad Prism 8.0 software (GraphPad Software, San Diego, CA, USA). All tests were two-sided, and differences were considered significant when *p* < 0.05. Comparisons of categorical variables were performed using a chi-square test or Fisher’s exact test, as appropriate. Comparisons of quantitative variables were performed using non-parametric tests, as the distribution of normality was variable within different immune biomarkers and TMB. Accordingly, Mann–Whitney and Kruskal–Wallis tests were used to analyze differences in immune cell counts and TMB.

## 5. Conclusions

In this study we analyzed the immune context of sporadic and hereditary FAP-related lesions along the colorectal ACS. Our findings showed that colorectal carcinogenesis is characterized by the establishment of a progressively “colder” immune microenvironment, with a gradual loss of adaptive immune cell counts along the colorectal ACS. These dynamic modifications of the tumor microenvironment do not appear to be related to neither the loss of tumor cell immunogenicity nor to the establishment of an immunosuppressive microenvironment. Rather, the overall decrease in tumor-infiltrating immune cells along the colorectal ACS may be better explained by a sustained antigen stimulation and chronic inflammation that leads, paradoxically, to progressive immune cell dysfunctionality and exhaustion.

## Figures and Tables

**Table 1:** Clinicopathological characteristics of sporadic and Familial Adenomatous Polyposis (FAP) colorectal cancer cases.

Feature	Sporadic ADCs *n* = 14	FAP ADCs *n* = 7
	Median value (range)	Median value (range)
**Age**	62 (35–82)	51 (45–63)
**Gender**	n (%)	n (%)
Male	8 (57.1)	3 (42.9)
Female	6 (42.9)	4 (57.1)
**WHO classification** (2019) [21]		
ADC NOS	14 (100)	7 (100)
**pT stage** (AJCC 8th Ed) [22]		
pT1	5 (35.7)	2 (28.6)
pT2	4 (28.6)	1 (14.3)
pT3	4 (28.6)	3 (42.8)
pT4	1 (7.1)	1 (14.3)
**pN stage** (AJCC 8th Ed) [22]		
pN0	10 (71.4)	4 (57.1)
pN1a	2 (14.3)	1 (14.3)
pN1b	2 (14.3)	2 (28.6)
**Grading**		
Low-grade	13 (92.9)	7 (100)
High-grade	1 (7.1)	0 (0)

ADC, invasive adenocarcinomas; NOS, not otherwise specified.

## Data Availability

The data presented in this study are available in the article itself.
